# Mitochondria—A billion years of cohabitation

**DOI:** 10.1371/journal.pbio.2002338

**Published:** 2017-03-30

**Authors:** Roland G. Roberts

**Affiliations:** Public Library of Science, Cambridge, United Kingdom

An engulfment of a small bacterium by a larger archaeon more than a billion years ago resulted not in death, but in one of the most successful partnerships on earth. As you sit there, each of your cells (apart from your red blood cells) contains hundreds or thousands of the descendants of that bacterium, still earning their keep as part of that endosymbiotic deal struck in some ancient pool. However, a billion years of intimacy has blurred the distinction between these partners and shaped them irrevocably.

The host cell provides protection and food to the mitochondrion, and supplies all but a handful of the hundreds of proteins needed to run its affairs (this handful are encoded by a tiny rudimentary mitochondrial genome, a mere vestige of its bacterial forebear). The deal cuts both ways, though, and as a quid pro quo the cell delegates a number of rather grubby tasks to these tiny organelles. The most obvious of these is that the mitochondria make most of the cell’s energy currency, in the form of ATP. But they also help store and regulate calcium, synthesize and degrade specific chemicals, and take part in the cell’s decision to commit suicide.

Like many factories, mitochondria are dangerous places with unpleasant chemicals, and handling the cell’s dirty work takes its toll on the organelles, threatening their integrity and that of their pared down genomes. This makes the quality control of mitochondria in the face of such damage a crucial issue in evolution, disease, and aging.

**Fig 1:**
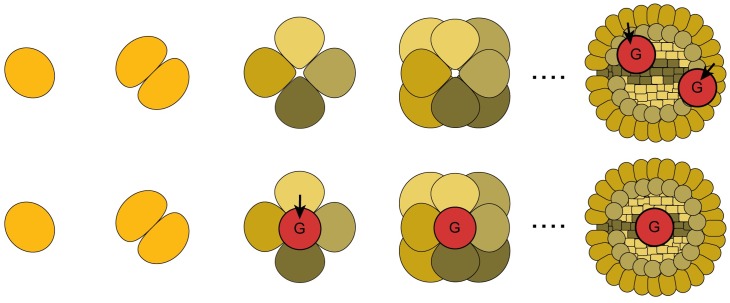
To sequester or not to sequester. As zygotes develop to adulthood (left to right), the germline (red) of different organisms can either arise late in development (top row) or be sequestered early on (bottom row). A recent study [1] suggests that the choice of strategy is driven by mitochondrial mutation rate. *Image credit*: *journal*.*pbio*.*2000410*.

A recent study in *PLOS Biology* by Nick Lane and colleagues [1] suggests that the need to pass on high-quality mitochondria to our offspring has determined how organisms choose which cells will contribute to the next generation. Plants can generate gametes from many parts of their bodies, even relatively late in development, producing flowers from recently grown stems. However, humans—and many other animals—have elected to set aside (“sequester”) a distinct “germline” at an early stage of development, keeping it safe for when it’s needed (Fig 1). Why the difference in strategy?

These authors use a mathematical model to argue that a major determinant of this decision—to sequester or not—is down to selection for mitochondrial quality. Each time a cell divides, mutations arise and mitochondria are partitioned between the daughter cells (and the tissues they form), generating a population with variable fitness on which selection can act. Plants and primitive animals, which have low mutation rates, can afford selection to occur over many rounds of somatic cell division en route to gamete formation. The evolution of metabolically expensive lifestyles like predation (and its corollary—evasion) in the Cambrian explosion, however, entailed substantially increased mitochondrial mutation rates. The authors show that this shifts the balance, favouring the early set-aside of the cells from which breeding occurs (the germline), and thereby limiting the number of rounds of cell division incurred.

In addition, where transmission of mitochondria is a job entrusted wholly to the mother (as in humans), this has further intriguing consequences. Here the opportunity for selection on mitochondrial quality is further reduced by the large number of mitochondria in each egg; this is addressed by a profligate process in which a huge excess of oocytes is produced through additional rounds of cell division (reinstating the variation), followed by ruthless (but random) culling.

This vulnerability of the mitochondrial genome can have a more tangible and immediate effect on our lives. Two recent *PLOS Genetics* papers survey the burden of mutations borne by mitochondrial genomes both in the germline and the soma. A survey of mitochondrial DNA from more than 500 human tumours [2] reveals a compendium of more than 600 somatic mutations. The authors find that although in cancer the rules of evolution are rewritten to prioritise the cell over the organism, selection still strongly favours a functioning mitochondrion. By contrast, a search for the germline mutations that cause congenital mitochondrial disorders [3] reveals the extent to which proteins encoded by the nuclear genome contribute to mitochondrial function. The authors screened the mitochondrial and nuclear genomes of 142 patients, finding clear causal mutations in about a third of cases (mostly in nuclear genes), and suspicious variants in more than half of the remainder.

Although the textbook picture of a eukaryotic cell still tends to depict mitochondria as discrete jelly bean-shaped objects, in reality they’re rather fluid organelles, dynamically fusing to form networks and then splitting off again, allowing the destruction of damaged mitochondria by mitophagy and their replacement by biogenesis. The authors of two *PLOS ONE* papers set out to model (in very different ways) this complex set of processes to see how useful mitochondrial function can be maintained in an aging body [4] and how day-to-day mitochondrial responses to energetic demands and stress are managed [5].

Not only do mitochondria form their own dynamic network, but they have also evolved to interact intimately with other systems in the cell; none more intimately than the endoplasmic reticulum (ER), a pervasive tubular network within the cytoplasm. So close is this relationship, in fact, that a recent *PLOS Biology* paper shows that mutations in an ER protein (wolframin) probably cause a human neurological disease (Wolfram syndrome) via their effects on mitochondrial dynamics [6]. An *eLife* paper stresses the importance of the ER-mitochondrial pas-de-deux for regulating apoptosis, a key mitochondrial role; the authors found that the regulated bridging of ER and mitochondria by a protein called IRBIT helps trigger influx of calcium ions from ER to mitochondria, kick-starting apoptosis [7]. Calcium management also underlies an intriguing cell type-specific function of mitochondria in neurons, a *PLOS Biology* paper reveals—cortical synapses with associated mitochondria have distinct presynaptic neurotransmitter release properties because the mitochondria mop up excess calcium [8].

A *Scientific Reports* paper shows that calcium isn’t the only currency at trading points between these organelles; the membrane lipid phosphatidylserine is also trafficked from ER to mitochondria, dependent on the ER-mitochondria encounter structure (ERMES) [9]. The degree of complexity of ER-mitochondrial interactions is reflected in the steady stream of additional players recognized, such as the roles described for the small GTPase Sar1 [10] and the multi-membrane-spanning protein VMP1 [11] in two recent *PLOS ONE* papers.

So after one billion years of living together, we have subjugated a free-living bacterium, turning it into a domesticated organelle. But it has also shaped us, and it’s sobering to think that if it weren’t for mitochondria then instead of neatly setting aside our germlines in two tidy gonads within a couple of months of conception, we might be sprouting them all over our bodies as we reach sexual maturity.

For more detailed reading please see the associated PLOS Collection [12].
